# Event‐related potential patterns of selective attention modulated by perceptual load

**DOI:** 10.1002/brb3.2907

**Published:** 2023-02-14

**Authors:** Zhuo Chen, Yun Qin, Maoqin Peng, Wei Zhao, Xuqian Shi, Danwei Lai, Erwei Yin, Ye Yan, Dezhong Yao, Tiejun Liu

**Affiliations:** ^1^ The Clinical Hospital of Chengdu Brain Science Institute, MOE Key Lab for Neuroinformation University of Electronic Science and Technology of China Chengdu China; ^2^ Sichuan Institute for Brain Science and Brain‐Inspired Intelligence Chengdu China; ^3^ The Defense Innovation Institute Academy of Military Sciences Beijing China

**Keywords:** ERP, perceptual load, response competition, selective attention

## Abstract

**Introduction:**

A high perceptual load can effectively prevent attention from being drawn to irrelevant stimuli; however, the neural pattern underlying this process remains unclear.

**Methods:**

This study adopted a perceptual load paradigm to examine the temporal processes of attentional modulation by incorporating conditions of perceptual load, distractor‐target compatibility, and eccentricity.

**Results:**

The behavioral results showed that a high perceptual load significantly reduced attentional distraction caused by peripheral distractors. The event‐related potential results further revealed that shorter P2 latencies were observed for peripheral distractors than for central distractors under a high perceptual load and that a suppressed compatibility effect with increasing load was reflected by the P3 component.

**Conclusion:**

These findings suggested that (1) P2 and P3 components effectively captured different sides of attentional processing modulated by load (i.e., the filter processing of the object and the overall attentional resource allocation) and (2) response patterns of selective attention modulated by perceptual load were influenced by eccentricity. Our electrophysiological evidence confirmed the behavioral findings, indicating the neural mechanisms of attentional modulation.

## Graphical abstract

This research measured the effects of perceptual load on modulating selective attention from a new insight at later electrophysiological stages. In the current study, the P2 and P3 components were associated with different phases of an attention‐modulated process, corresponding to object filtering and overall attentional resource allocation, respectively. Overall, these findings provide electrophysiological evidence for attention quantification and further the understanding of attentional modulation.

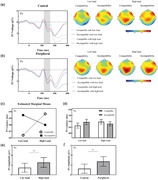

## 1 INTRODUCTION

Selective attention helps individuals focus on task‐relevant stimuli while ignoring irrelevant stimuli, which is critical when individuals are faced with complex tasks in daily life. Much work focusing on how much attention to task‐relevant stimuli can be guaranteed in the presence of task‐irrelevant potential distractors through various paradigms has been done in the past (e.g., visual search task, perceptual load task, and four‐choice localization tasks) (Fritzsche et al., 2011; Schindler et al., 2021; Watson et al., 2020). However, the neural mechanisms of selective visual attention and the potential factors that may influence it remain unclear and have gained great interest.

Perceptual load theory suggests that the ability to focus attention and reject distractors depends on the level of perceptual load required for the current processing (Lavie, 1995, 2005, 2010; Lavie et al., 2004). Specifically, under a low perceptual load, the resources remaining after target task‐related processing overflow automatically, and then distractors are processed. Under a high perceptual load, the processing of the target stimulus exhausts all attentional resources, and available resources are lacking for processing distractors. Recently, the level of the perceptual load has become an important factor to consider in selective attentional processing (Geden et al., 2018; Neokleous et al., 2016; Tyndall et al., 2018).

Functional magnetic resonance imaging (fMRI) studies showed a load‐dependent selective attention mechanism that was enhanced by target‐related brain activity as the perceptual load increased accompanied by a decrease in distractor‐related activity, which was under the control of the frontoparietal network associated with spatially directed attention (Pinsk et al., 2004; Rees et al., 1997). Electroencephalography (EEG) results showed that early event‐related potential (ERP) components could reflect selective attentional responses involving a perceptual load, suggesting that the perceptual load had already influenced information flow during the initial stages of visual cortical processing (Fu et al., 2010; Handy & Mangun, 2000; Handy et al., 2001). However, a recent systematic evaluation suggested that ERP effects of load‐attenuating distractor processing were more reliable at a slightly later stage than the mixed results in the early attention selection process (Brockhoff et al., 2022). For example, deeper features increased the anterior P2 amplitudes, which might reflect the process of visual feature detection and attentional selection (Cao et al., 2020; Correll et al., 2006; Nikolaev et al., 2008). It was further suggested that the P2 component was sensitive to attention processing and load, with larger amplitudes observed in the processing of emotional distractors under low loads (Doallo et al., 2006). In parallel, the parietal P3 component was used as a valid measure of voluntary attention resource allocation and indicated effective responses to the demand for attentional resources under a perceptual load (Harris et al., 2019). In light of previous efforts, focusing on the activity of the later stages (i.e., P2, P3) appeared to be a promising way to better understand the neural mechanisms in attentional processing.

In addition to the objective recording of experiments, self‐observation contributes to a more comprehensive understanding of attention. A scale that appears to do this is the Mindful Attention Awareness Scale (MAAS; Brown & Ryan, 2003), which measures the perceived degree of attentiveness in different contexts or is a stable psychological trait when used outside of attentional training situations (Brown et al., 2015, 2011). The MAAS is considered a prospective predictor of not only self‐reported attention‐related errors but also of a behavioral measure of the ability to sustain conscious awareness of attention (Cheyne et al., 2006). Studies exploring the relationship between the MAAS and attentional control showed that individuals diagnosed with attention‐deficit hyperactivity disorder who were deficient in attentional control skills scored lower on the MAAS than controls. Of note, the degree of attention lapses was negatively correlated with MAAS scores, implying that higher MAAS scores may be associated with better attentional control (Keith et al., 2017). Thus, we included the MAAS as a method of evaluating concentration ability from a first‐person perspective, with high total scores reflecting a strong ability to focus attention.

This study aimed to assess neural responses at a later stage during attentional processes modulated by different perceptual loads. In addition, we included eccentricity as a potential factor and analyzed the neural response patterns with which it was involved (Beck & Lavie, 2005). We hypothesized that attention would be modulated by perceptual load and that the P2 and P3 components would have specific representations in attentional processing. Specifically, attention to nontargets may behaviorally decrease (measured by compatibility effects) as load increases (Lavie, 2005, 2010). In terms of neural responses, the latency or amplitude of the P2 component may vary over the load and be potentially influenced by eccentricity, and the P3 component may tend to respond more to the overall allocation of attentional resources.

## 2 METHOD

### 2.1 Participants

Thirty‐one right‐handed males from the University of Electronic Science and Technology of China were recruited (mean age ± SD = 22.29 ± 1.81 years). The sample size was derived by G*Power (1 group, 4 measurements, *α* = 0.05, and a 0.5 correlation among repeated measures) using a medium effect size (0.25) (Kang, 2021), suggesting that 24 subjects would achieve an acceptable statistical power (0.8) (Faul et al., 2009). All participants had normal or corrected‐to‐normal vision, had no personal psychiatric or neurological disorders, had not taken other drugs that acutely or chronically harmed the central nervous system, and had not consumed or inhaled psychostimulant foods and gases during the experimental period. Participants were required to complete the MAAS before the experiment; they also provided written informed consent and received appropriate monetary compensation after the experiment. This experiment was performed according to the principles of the Declaration of Helsinki and the guidelines established by the University of Electronic Science and Technology of China.

### 2.2 Apparatus and stimuli

Based on previous studies, our experimental design was a combination of the perceptual load paradigm and EEG (Green & Bavelier, 2003, 2006; Proksch & Bavelier, 2002). E‐Prime 2.0 software (Psychology Software Tools, Inc., Pittsburgh, PA, USA) was used to generate the stimuli on an Intel Core i5‐10400 CPU at 2.90 GHz. Stimuli were presented on a 20‐inch monitor with a video graphics card, a refresh rate of 60 Hz, and a resolution of 1600 × 900 pixels. Participants viewed displays on the screen at a distance of 57 cm.

**FIGURE 1:**
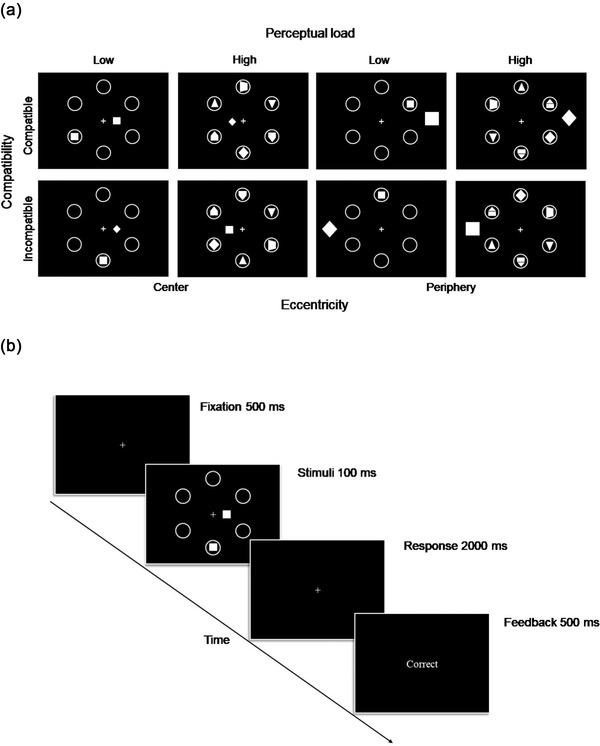
(a) Perceptual load stimuli. Each graphical trial consisted of three items: the target, a distractor, and a filler. The perceptual load was determined by the number of fillers, eccentricity was determined by the distractor location, and compatibility was determined by the relationship between the target and the distractor. (b) The experimental procedure.

As shown in Figure 1a, one trial contained three types of items (the target, a filler, and a distractor) in white against a black background. Each item consisted of a separate set of graphs: the target set included a square and a diamond; the distractor set was composed of a square, a diamond, and a slender ellipse; and the filler set consisted of a pentagon, an upside‐down pentagon, a lateral trapezoid, and a triangle that was pointed either up or down. Only one target and one distractor were displayed each time, and the number of fillers was randomly set to 0, 1, 3, or 5, while the target and fillers were displayed in six identically sized circular frames to facilitate localization (Maylor & Lavie, 1998). The distractor appeared in one of four possible locations depending on the eccentricity, which was central (0.5° to the right or left of the fixation icon) or peripheral (4.2° to the right or left of the fixation icon).

The average size of the target and the filler was 0.6° vertically and 0.4° horizontally. In addition, aiming to elicit the same amount of activity in the primary visual cortex for both eccentricity conditions, the size of the central distractor was 0.3° vertically and 0.2° horizontally, and the peripheral distractor was 0.9° vertically and 0.5° horizontally, adjusted by the cortical magnification factor (Rovamo & Virsu, 1979). Six circular frames were placed in a ring around the fixation icon at a distance of 2.1°, the same as the distance between the center of each two adjacent circular frames.

Regarding the experimental design, the compatibility depended on the relationship between the distractor and the target: when the distractor was an ellipse, the relationship was natural; when the distractor was the same as the target, the relationship was compatible; and when these two were opposite, the relationship was incompatible. These three conditions were randomly presented in equal numbers within each block. We measured the degree of attentional distraction by compatibility effects, defined as the extent to which response‐incompatible distractors interfered with task performance relative to response‐compatible distractors, that is, incompatible minus compatible mean reaction times (RTs). Except for the target within a specific frame, the number of fillers in the remaining circular frames corresponded to the low‐load condition (zero or one filler) or high‐load condition (three or five fillers). According to the position where the distractor appeared relative to the inside or outside of the large circle formed by six circular frames, eccentricity was classified as either a central position (inside the circle, bilateral) or a peripheral position (outside the circle, bilateral).

For targets, distractors, and fillers, their parameters, including the shapes, positions, and relative placements, were counterbalanced for each load level and presented randomly within each block. With the position (6) and shape control (2) of the target, 48 frames per block were suitable to demonstrate the full balance of the perceptual load (4), distractor compatibility (3), and distractor eccentricity (4).

### 2.3 Procedure

Each trial started with a 500‐ms fixed cross in the center of the screen, followed by a 100‐ms graphic presentation where subjects were required to identify the shape of the target object as soon as it appeared. After 2 s, a feedback interface, in which participants were to indicate their responses, including correct responses, incorrect responses, or untimely responses, was presented in text form for 500 ms (see Figure 1b). Subjects were to judge the shape of the target object as it was presented each time and ignore irrelevant stimuli with the right index and middle finger placed on two keys that they would use to indicate their responses (key 1 on the numeric keypad corresponded to the square target, and key 2 corresponded to the diamond target). The participant's goal in the task was to respond as quickly and accurately as possible and to achieve a 90% correct rate.

Subjects initially performed the practice phase (two blocks, 96 trials), and after they understood the task requirements, they were able to complete the formal experiment (12 blocks, 576 trials). In the formal experiment, subjects received information about the RT and accuracy of the previous block at the end of each block, at which point they decided whether to take a short break to gain sufficient energy.

### 2.4 EEG acquisition and preprocessing

The behavioral and EEG data were collected simultaneously in a dark, quiet, and electromagnetically shielded room. According to the international 10–20 system, the 32‐channel EEG signals were recorded by using a Biosemi ActiveTwo system (BioSemi, Amsterdam, Netherlands) and digitized at a sampling rate of 2048 Hz. The online filter band was 0.16–100 Hz, the ground was replaced by the driven right leg (DRL) passive electrode, and electrode Fz was set as the reference channel.

To obtain reliable results, a series of EEG preprocessing procedures were adopted, including 0.1–30 Hz IIR bandpass filtering; resampling to 512 Hz; referencing to “infinity” zero (Tian & Yao, 2013; Yao, 2001); segmenting from −200 to 600 ms (time‐locked to stimulus onset) followed by a 200‐ms baseline correction; removing eye‐movement and blink artifacts based on the independent component analysis (ICA) method (the rejection rate of each subject was kept below 5%), which had been adopted by a series of studies with stable results (Koelstra et al., 2009; Koroma et al., 2020); and removing trials with artifacts (moving window peak‐to‐peak method with a 200 msec moving window, a 100 msec window step, and a 65 μV threshold, resulted in a rejection of an average 5.2% of trials).

### 2.5 Data analysis

The following analysis was based on previous studies (Green & Bavelier, 2003, 2006; Lavie, 2010). First, trials with incorrect responses were excluded. RTs (greater than 300 ms and less than 1800 ms) and error rates were included separately in a three‐factor within‐subject analysis of variance (ANOVA) as a function of perceptual load (low vs. high), compatibility (compatible vs. incompatible), and eccentricity (central vs. peripheral). Natural distractors were not included in the current analysis as they were not the main focus of this study (Green & Bavelier, 2003, 2006).

Second, the mean ERPs for different conditions were obtained by averaging corresponding trials across subjects. We selected P2 at electrode Fz and P3 at electrode Pz as the main components based on previous studies and EEG topography (Brockhoff et al., 2022; Carlson, 2021; Polich, 2012). For latency, we adopted the “50% area latency” measure. This method was shown to work effectively for large components such as P3 waves, by averaging voltages of individual data points within a specified window and determining the specific point that divided the area under the curve into a 50% fraction (Kiesel et al., 2008; Luck & Hillyard, 1990). Then, we averaged the voltages at each point within a given time window to obtain more stable ERP amplitudes (Gan et al., 2020; Martens et al., 2006). Based on the grand average waveform, the specific time window for extracting waveforms was set from 170 to 270 ms for P2 and from 230 to 330 ms for P3. Third, the same three‐way ANOVA with a design of 2 (perceptual load: low vs. high) × 2 (distractor eccentricity: central vs. peripheral) × 2 (compatibility: compatible vs. incompatible) corresponding to the behavioral analysis was performed for the latencies and amplitudes of the P2 and P3 components, respectively.

Finally, the relations between P3 and behavioral responses were examined based on the Pearson correlation to better integrate neural responses with attention outcomes. We selected electrophysiological indicators of P3, including the amplitude and compatibility effect (the latency for incompatible conditions minus the latency for compatible conditions), and the behavioral responses, including the MAAS score and error rate, as the main components of interest and correlated them correspondingly in an exploratory analysis. In addition, because the compatibility effect of P3 latency was conceptually and temporally consistent with the compatibility effect of behavior, we performed a correlation analysis between them.

EEG data were analyzed by using MATLAB (MathWorks Inc. Natick, MA, USA), EEGLAB (Delorme & Makeig, 2004), and ERPLAB (Lopez‐Calderon & Luck, 2014). Statistical analysis of behavioral and EEG data was performed (Bonferroni correction, *p* < .05) with SPSS 22.0 software (SPSS Inc., Chicago, IL, USA). Additionally, Bayes factors (BF_10_) were estimated to reveal the strength of critical null effects by measuring the degree to which an alternative hypothesis was supported by the data relative to a null hypothesis.

## 3 RESULTS

### 3.1 Reaction times

**FIGURE 2:**
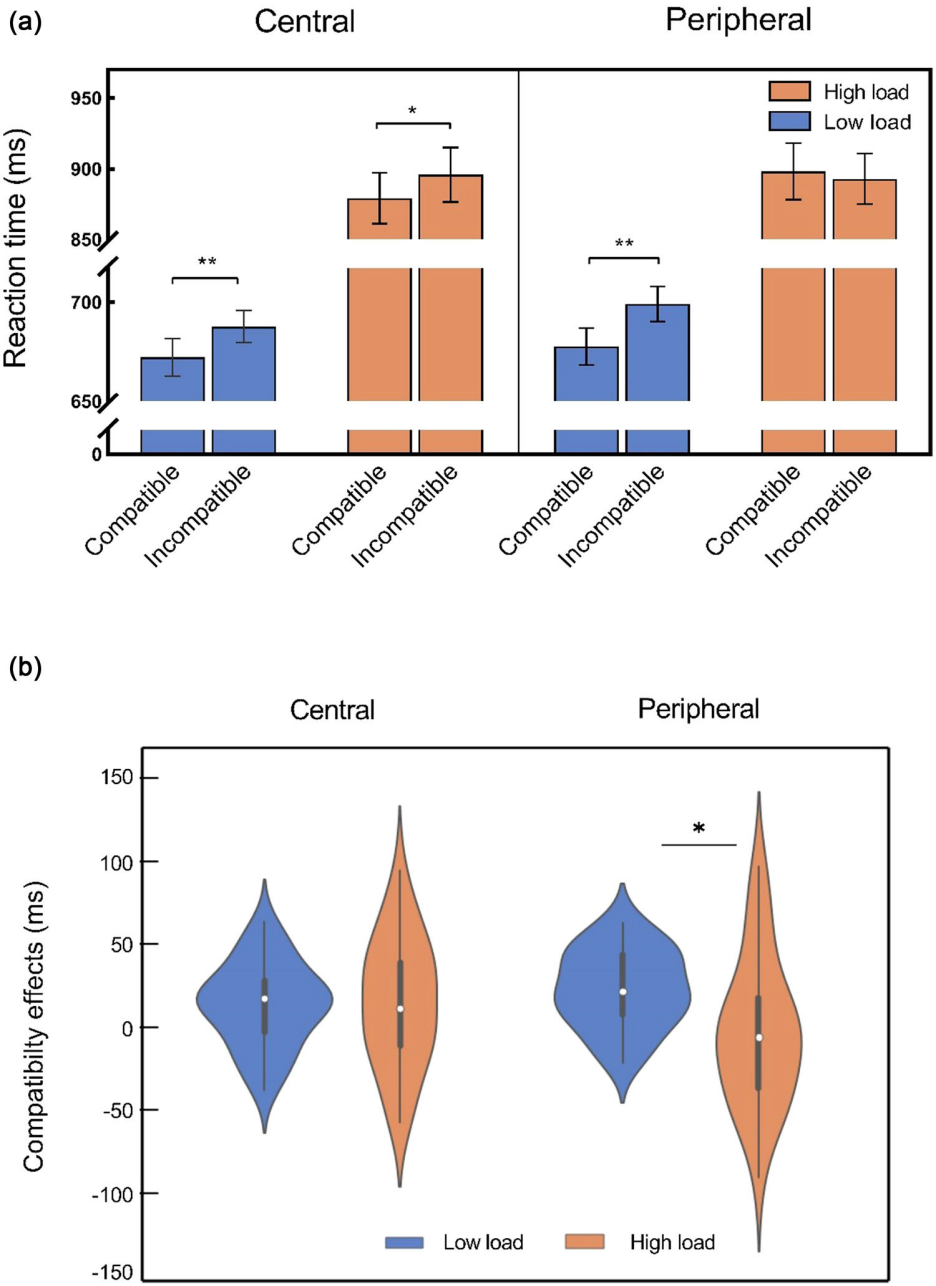
Behavioral results. (a) Perceptual load × compatibility × eccentricity interaction in reaction times (RTs). (b) The compatibility effect with perceptual loads at different eccentricity rates, suggesting that the compatibility effect for peripheral distractors decreased more significantly with increasing load but not for central distractors. Error bars refer to the standard error of the mean (SEM), *p < .05, **p < .01.

The results revealed a significant main effect of perceptual load [*F*(1, 30) = 253.40, *p* < .001, $\eta_{p}^{2}$ = 0.89], showing that the task became more difficult as the perceptual load increased (low load: 684.21 ms ± 8.48; high load: 891.47 ms ± 18.20). A significant main effect of compatibility was identified [*F*(1, 30) = 12.54, *p* = .001, $\eta_{p}^{2}$ = 0.30], demonstrating the effect of distractor compatibility on RT (compatible distractors: 781.81 ms ± 12.95; incompatible distractors: 793.87 ms ± 12.51). There was also a significant main effect of eccentricity [*F*(1, 30)= 9.70, *p* = .004,$\;\eta_{p}^{2}$ = 0.24], suggesting a general RT cost associated with the presence of stimuli in the periphery (vs. in the central region) (central distractors: 783.77 ms ± 12.55; peripheral distractors: 791.91 ms ± 12.82). The interaction effect between eccentricity and compatibility was marginally significant [*F*(1, 30) = 3.48, *p* = .072, $\eta_{p}^{2}$ = 0.10]. Exploratory analysis showed that central distractors produced greater compatibility response effects [*t*(30) = 4.29, *p* < .001, Cohen's *d* = 0.77] (Table S1). None of the other two‐way interactions (i.e., load and compatibility; eccentricity and load) were significant (all *p*s > .108). Of note, a perceptual load × compatibility × eccentricity interaction was observed [*F*(1, 30) = 6.19, *p* = .019, $\eta_{p}^{2}$ = 0.17], as shown in Figure 2a. To assess the three‐way interaction, a further 2 (load: low, high) × 2 (compatibility: compatible, incompatible) within‐subjects ANOVA was conducted for each distractor eccentricity. For the peripheral condition, the main effect of compatibility did not reach significance (*p* = .067); however, a main effect of load [*F*(1, 30) = 238.64, *p* < .001, $\eta_{p}^{2}$ = 0.89] and a load‐compatibility interaction emerged [*F*(1, 30) = 7.413, *p* = .011, $\eta_{p}^{2}$ = 0.20]. Post hoc analysis of paired *t* tests showed that under the low‐load condition, responses to incompatible distractors were significantly later than compatible distractors [*t*(30) = 5.00, *p* < .001, Cohen's *d* = 0.90]; under the high‐load condition, there was no significant difference between incompatible and compatible distractors [*t*(30) = −0.65, *p* = .523], indicating that a high perceptual load had a significant reduction in the compatibility effects of peripheral distractors compared to that of a low perceptual load (Figure 2b; Table S1). For the central condition, there were main effects of load [*F*(1, 30) = 237.58, *p* < .001, $\eta_{p}^{2}$ = 0.89] and compatibility [*F*(1, 30) = 18.37, *p* < .001, $\eta_{p}^{2}$ = 0.38], with high load (vs. low load) and incompatibility (vs. compatibility) contributing to an increase in RT (Table S1). However, no load‐compatibility interaction was found [*F* < 1, *BF*_10_ = .128], suggesting that increasing load resulted in no meaningful reduction in the compatibility effect of central distractors (Figure 2b; Table S1).

### 3.2 Error rates

Error rates revealed significant main effects of compatibility [*F*(1, 30) = 46.61, *p* < .001, $\eta_{p}^{2}$ = 0.61] and load [*F*(1, 30) = 267.47, *p* < .001, $\eta_{p}^{2}$ = 0.90] with fewer errors in the low‐load (4.84% ± 0.71) condition than in the high‐load (22.70% ± 1.51) condition and fewer errors in the compatible (11.93% ± 0.97) condition than in the incompatible (15.61% ± 1.18) condition. There was no main effect of eccentricity (*p* = .304) or other interaction effects (i.e., compatibility × eccentricity: *p* = .062; compatibility × load: *p* = .777; load × eccentricity: *p* = .695; compatibility × eccentricity × load: *p* = .418). See the additional descriptive information about error rates in Table S1.

### 3.3 Event‐related potential

#### 3.3.1 P2

**FIGURE 3:**
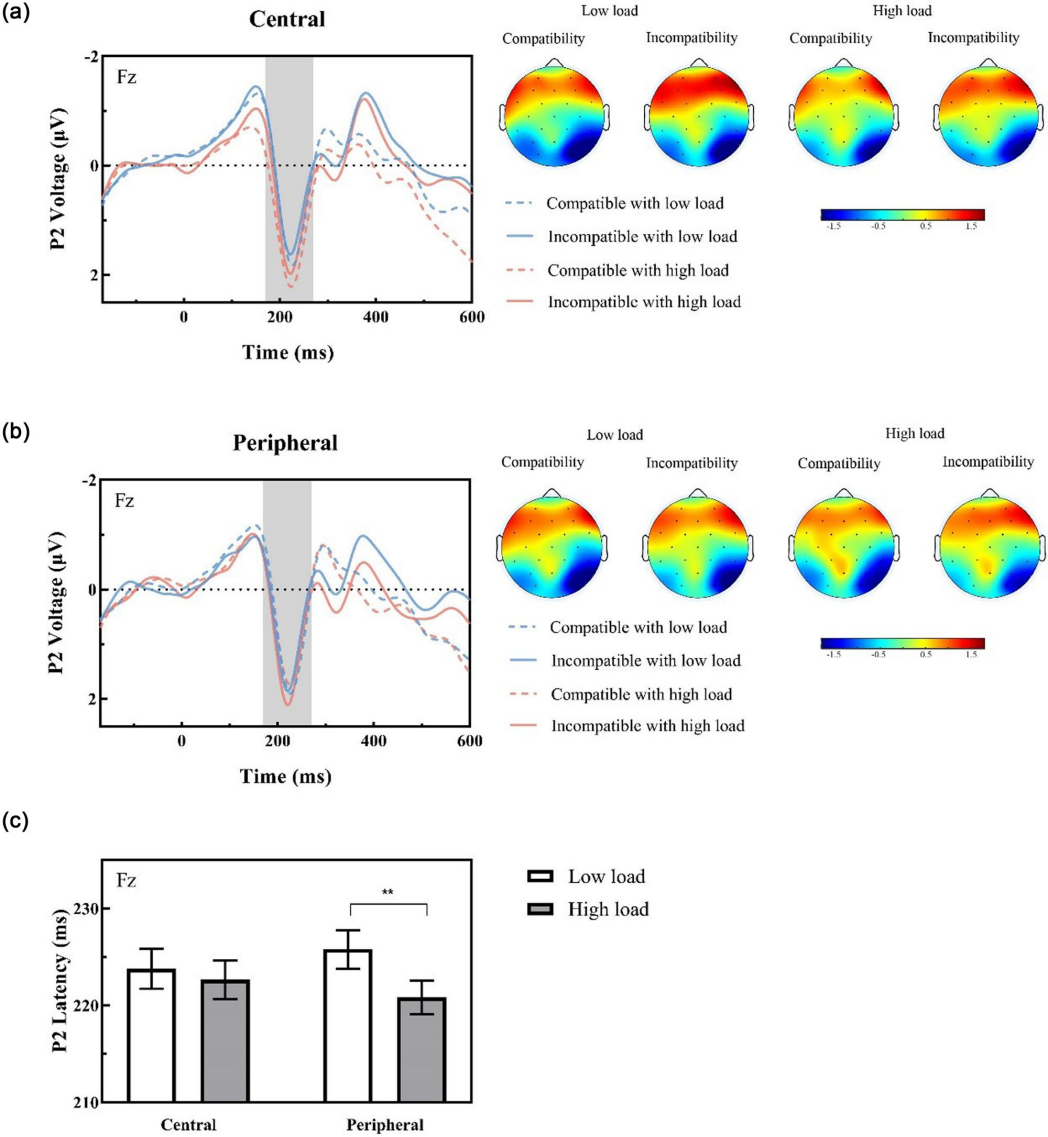
P2 data (left side) for central distractors (a) and peripheral distractors (b) under differential load levels at the Fz electrode with corresponding topographic maps (right side). (c) The peripheral trials reflected a significantly shorter latency in the high‐load condition than in the low‐load condition; however, this outcome was not observed in central trials. Topographic maps represented the average amplitude consistent with the P2 time window from 170 to 270 ms. Error bars refer to the SEM, **p < .01.

P2 latencies revealed no main effects of compatibility (*p* = .253) or eccentricity (*p* = .922) but a main effect of load [*F*(1, 30) = 5.36, *p* = .028, $\eta_{p}^{2}$ = 0.15] (low load: 224.79 ms ± 1.95; high load: 221.74 ms ± 1.76) and a significant interaction between load and distractor eccentricity [*F*(1, 30) = 4.81, *p* = .036, $\eta_{p}^{2}$ = 0.14]. Post hoc analysis by paired samples *t* tests suggested no significant difference between high‐ and low‐load conditions when a central distractor was present [*t*(30) = 0.71, *p* = .487, *BF*_10_ = .214] (Figure 3a); in peripheral trials, however, latencies caused by high perceptual load were significantly earlier than those under low‐load conditions [*t*(30) = 3.2, *p* = .003, Cohen's *d* = 0.57] (Figure 3b, see Figure 3c for bar graphs). None of the other two‐way interactions between compatibility and distractor eccentricity (*p* = .497) or between compatibility and load (*p* = .690) was significant. The three‐way interaction of load, distractor eccentricity, and compatibility was not significant [*F* < 1], indicating that the perceptual load detection differentially associated with eccentricity affected the compatibility relationship to the same extent (Table S2).

P2 amplitudes showed no main effects (eccentricity: *p* = .751; load: *p* = .290) or interactions (compatibility × eccentricity: *p* = .160; compatibility × load: *p* = .928; load × eccentricity: *p* = .116; compatibility × eccentricity × load: *p* = .257) other than a marginally significant effect of compatibility [*F*(1, 30) = 3.24, *p* = .082, $\eta_{p}^{2}$ = 0.10] with greater P2 amplitudes in the incompatible condition than in the compatible condition (compatible distractors: 0.92 μV ± 0.30; incompatible distractors: 1.16 μV ± 0.29). For more descriptive information about the P2 component, see Table S2.

#### 3.3.2 P3

**FIGURE 4:**
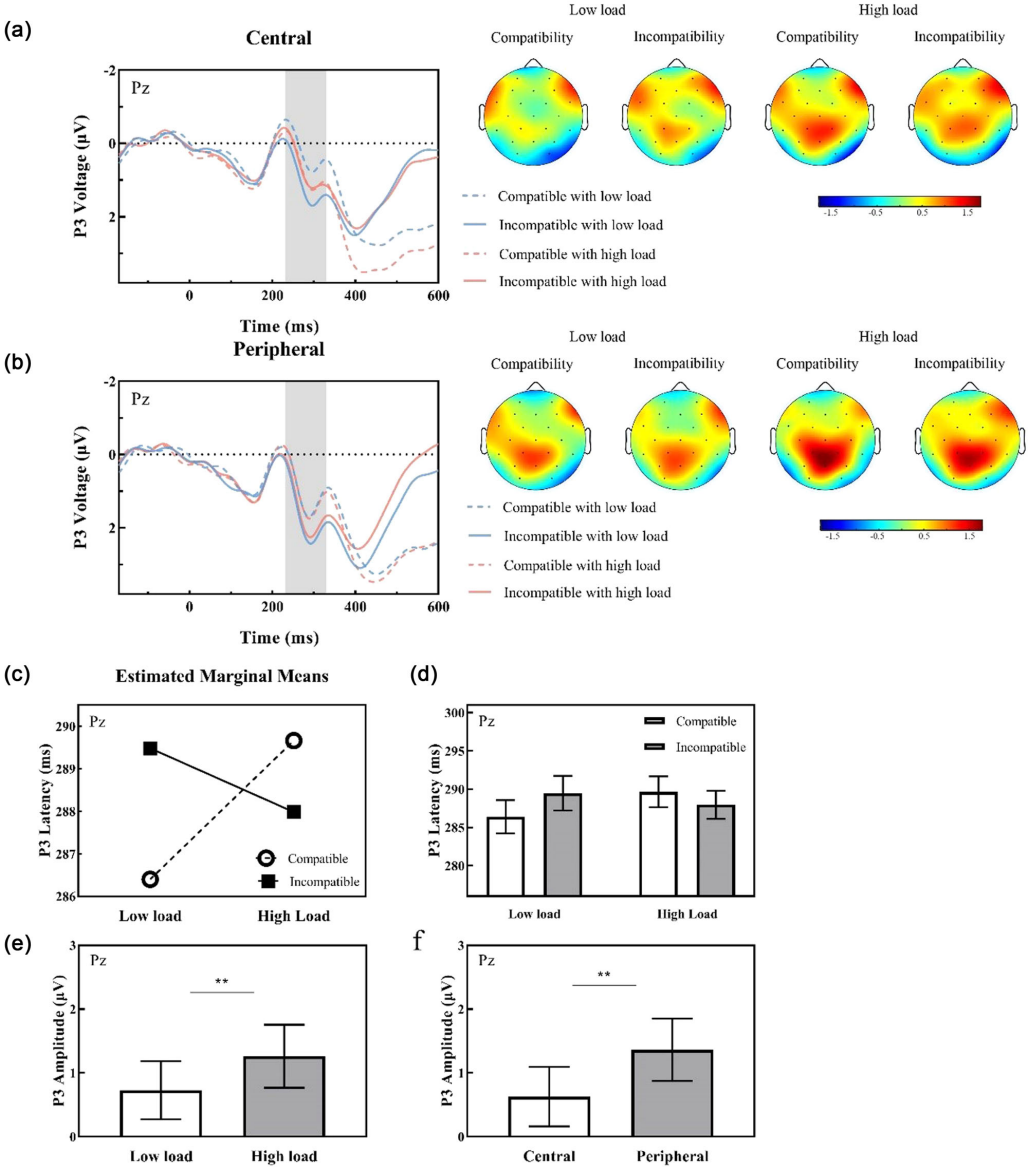
P3 data (left side) and corresponding topographic maps (right side) with load and compatibility conditions for central distractors (a) and peripheral distractors (b) at electrode Pz. (c) Estimated marginal means of P3 latency by load and compatibility. (d) As the perceptual load increased, incompatible latencies were ahead of compatible latencies, reflecting the change in compatibility effects under load manipulation. P3 amplitude for load (e) and eccentricity (f). Both peripheral distractors (vs. central distractors) and high load (vs. low load) induced a significant increase in P3 amplitude. Topographic maps represented the average amplitude consistent with the P3 time window from 230 to 330 ms. Error bars refer to the SEM, **p < .01.

The results for P3 latencies revealed no significant main effects (compatibility: *p* = .535; eccentricity: *p* = .647; load: *p* = .466) or interaction effects related to eccentricity (compatibility × eccentricity: *p* = .068; load × eccentricity: *p* = .559; compatibility × eccentricity × load: *p* = .969) (see Figure 4a,b for P3 waveforms). However, there was an interaction between compatibility and perceptual load [*F*(1, 30) = 5.68, *p* = .024, $\eta_{p}^{2}$ = 0.16], indicating that the compatibility effect decreased with increasing load (see Figure 4c,d). This effect was more likely driven by a combination of perceptual load and compatibility, as post hoc analyses showed no significance (all *p*s > .093).

P3 amplitudes revealed main effects of eccentricity [*F*(1, 30) = 21.80, *p* < .001, $\eta_{p}^{2}$ = 0.42] and load [*F*(1, 30) = 13.87, *p* < .001, $\eta_{p}^{2}$ = 0.32], indicating that peripheral distractors (vs. central distractors) and high load (vs. low load) draw considerably more attentional resources (peripheral distractors: 1.36 μV ± 0.49, central distractors: 0.63 μV ± 0.46; high load: 1.26 μV ± 0.50, low load: 0.73 μV ± 0.45; see Figure 4e,f). None of the main effects of compatibility (*p* = .819) or other interactions were significant (compatibility × eccentricity: *p* = .768; compatibility × load: *p* = .177; load × eccentricity: *p* = .445; compatibility × eccentricity × load: *p* = .320). More descriptive information on the P3 component is provided in Table S3.

### 3.4 Correlation between ERP and behavioral parameters

**FIGURE 5:**
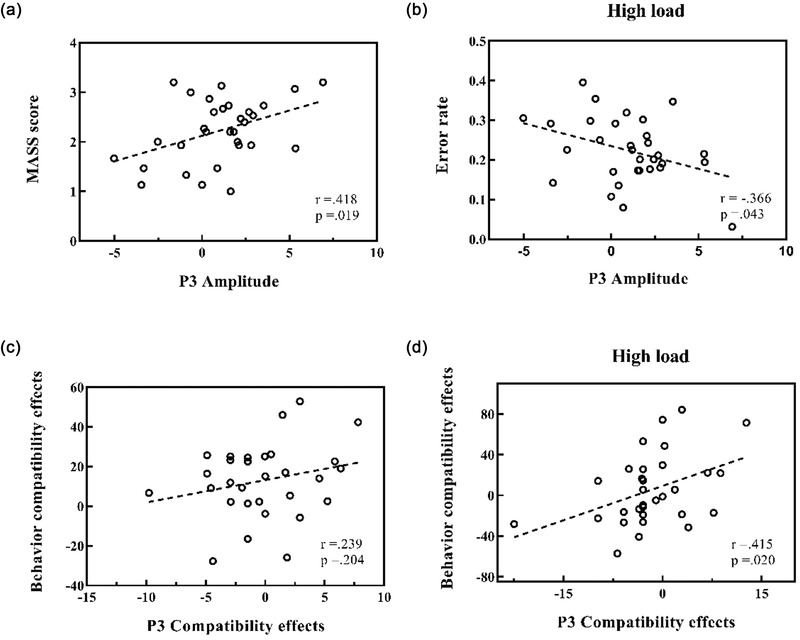
Plots showing the correlation of individual participants’ event‐related potential (ERP) measures with their behavior according to the relationship between P3 amplitude and Mindful Attention Awareness Scale (MAAS) score (a), P3 amplitude and error rate under high‐load conditions (b), compatibility effects between P3 latencies and behavior (c), and compatibility effects between P3 latencies and behavior under high‐load conditions (d). (a) and (b) show that the P3 amplitude is related to the individual's current level of attention and task performance under high‐load conditions. (c) and (d) indicate that the compatibility effects, a measure of the number of attentional resources, have the same trend between response time and P3 latencies, being more pronounced with higher attentional demands.

P3 amplitudes were positively correlated with MAAS scores (*r*_31_ = .418, *p* = .019, Figure 5a) and negatively correlated with error rates under high‐load conditions (*r*_31_ = –0.366, *p* = .043, Figure 5b) while not detected at low‐load conditions (*p* = .180). Regarding the P3 latency, there was a trend of a positive correlation between P3 compatibility effects and behavioral compatibility effects (*p* = .204, Figure 5c). Furthermore, the exploratory analysis revealed that this trend was sensitive to the perceptual load level, with a significant strong positive correlation specific to high‐load conditions between P3 compatibility effects and behavioral compatibility effects (*r*_31_ = 0.415, *p* = .020, Figure 5d) compared to low‐load conditions (*p* = .321). There was no significant correlation between P3 compatibility effects and MAAS scores (*p* = .733) or error rates (*p* = .055).

## 4 DISCUSSION

This study focused on electrophysiological markers of attentional modulation of visual processing and revealed the distinct roles of P2 and P3 in the modulation of attentional processes by perceptual load. Additionally, this study identified load‐modulated differences in selective attention associated with eccentricity and corresponding neural response patterns.

From a behavioral perspective, we verified that the attention resources differed in how they were modulated by perceptual load depending on different eccentricity rates (Green & Bavelier, 2006). Beck and Lavie found that fixation distractors produced stronger interferences (vs. peripheral distractors) by manipulating the position of distractors and further demonstrated the robustness that the information prioritization at fixation was unaffected by various other manipulations (Beck & Lavie, 2005). On this basis, we controlled the stimulus size to make the central and peripheral distractors equivalent in terms of retinal visual acuity under varied loading levels (Daniel & Whitteridge, 1961) and, interestingly, found that distractors in the central and peripheral areas produced different attentional trends with load changes. Specifically, as the load increased, the attentional distraction elicited by peripheral distractors decreased significantly compared to the change in the compatibility effects of central distractors that were modulated by the load, suggesting that the advantages of foveal attention were robust regardless of the load level and that peripheral attention was manipulable with respect to perceptual load. It was possible that since the attentional resources available to humans are limited, the tighter attentional resources that developed as perceptual load increased might preferentially support central attentional processing due to the attentional priority of the fovea itself (Calvo & Castillo, 2005). As a result, the attentional resources for central stimuli were relatively abundant, along with weakened attentional processing in the peripheral visual field. An alternative viewpoint suggested that the salience of the distractor may influence the allocation of focal attention (Schubö, 2009). The differences in the compatibility effect in the peripheral high‐load condition may be due to a reduction in distractor salience compared to that in the low‐load condition, where distractors may be more salient and therefore more likely to induce a spatial shift of attention. If the distractor salience and a spatial shift of attention played a major role, the central distractor was less likely to be attended than the peripheral distractor because it was differentially downsized according to the cortical magnification factor (central size: 0.3° vertically and 0.2° horizontally; peripheral size: 0.9° vertically and 0.5° horizontally, see Figure 1a). However, we found that the compatibility effect in the central high‐load condition was significantly larger than that in the peripheral high‐load condition [*t*(30) = 2.55, *p* = .016; central high‐load: 16.54 ms ± 7.06, peripheral high‐load: −5.25 ms ± 8.13]. Overall, these findings were more prone to reflect changes in the distribution of attentional resources under high and low loads.

To the best of our knowledge, the present study was the first to combine the perceptual load paradigm and ERP measurements to examine attentional processing under central/peripheral distractor manipulations. We chose perceptual load as the main comparison viewpoint to explore the relevant neural patterns under load variation, which was further identified by distractor eccentricity. The anterior P2 was shown to reflect a transdimensional feature detection process and some levels of higher order processing (Federmeier et al., 2005; Luck & Hillyard, 1994), and it has recently been proposed as a more integrated and extensive real‐time attentional marker (i.e., “gate‐keeper,” GK) (Perrone‐Bertolotti et al., 2020). This GK performance in late attentional selection occurs within 250 ms, responding to all incoming stimuli and holding the necessary information (Sawaki & Luck, 2010). It appears from the results that the P2 affected by the load manipulation was probably a marker of late attentional filtering; in particular, latency in the high‐load condition was significantly earlier than that in the low‐load condition. For an exploratory analysis, we analyzed P2 latencies of this effect by moving the acquisition point from Fz to electrodes F3 and F4 over the left/right inferior frontal sulcus (IFS), a location identified as a responsive brain region for GK via intracerebral EEG (Perrone‐Bertolotti et al., 2020). We found the same GK responses within 250 ms, as well as the main effects of load similar to Fz (see Figures S1 and S2), suggesting a relatively stable and faster response to high loads and unbiased lateralization of the broader GK mechanism distributed in prefrontal regions as detected by scalp EEG. The “GK” is suggested to have an integrative mechanism that determines the final impact of the incoming stimulus on the global brain through bottom‐up (stimulus‐driven features) and top‐down (expectations associated with the ongoing cognitive task) information. In our study, the mechanism of faster detection in the high‐load condition by the GK was helpful and efficient for subsequent controls and the eventual allocation of attentional resources.

In addition, we found similar responses to task‐related processes, as different distractor‐target relationships (compatible and incompatible) induced the P2 component, which was consistent with the actions that should be taken as an efficient “GK,” as it would handle all salient stimuli (potential threats should remain noticed) to save cognitive resources for task‐related items. Interestingly, there was an interaction between eccentricity and perceptual load, whereby P2 latency was shorter in the peripheral high‐load condition than in the peripheral low‐load condition with no considerable change in central distractors. This was in agreement with the direction of our behavioral results, probably a consequence of the combination of both the perception of the optic rod cells in the periphery of the retina and the tendency to focus on the central visual field. However, this finding should be interpreted with caution, as unnoticed changes may have an effect on the ERP signal. We did the material balance to the best of our ability, but we could not completely rule out a possible material effect because of the need to include different amounts of filler items for separating low and high loads, and this outcome needs to be substantiated through replication.

Brain imaging studies using fMRI implicated an execution function network involved in the top‐down control of attention (Giesbrecht et al., 2003; Witt et al., 2021), and several studies have further explored attentional processing through the parietal P3 as an electrophysiological indicator (Gan et al., 2020; Lin et al., 2018). Our results mainly showed that the compatibility effect of P3 latency decreased significantly as perceptual load increased and electrophysiologically confirmed a load‐modulated attention pattern similar to behavior (Lavie, 1995, 2005, 2010; Lavie et al., 2004). As revealed in previous studies, P3 was highly sensitive to the processing capacity of attentional resources required in the task (Herrmann & Knight, 2001; Polich, 2012), and P3 latencies were found to be closely linked to RTs, with earlier latencies indicating faster response execution and shorter response times (Doucet & Stelmack, 1999; Pfefferbaum et al., 1980). Furthermore, the parietal lobe was shown to play the role of a hub node in target selection to guide visual attention, and P3 could depict the deeper mechanism of this time‐varying brain network, reflecting top‐down attentional control through the difference between the response to the target and a distractor (Bisley & Goldberg, 2010; Li et al., 2016). Thus, what we observed by activation of this area may reflect the overall processing of attentional resources during higher cognitive processing as a result of the assessment of the current state; this reduced compatibility effects of P3 in attentional processing between high and low loads reflected the subsequent behavioral effects on performance. In addition, we observed that high‐load conditions (vs. low load) and peripheral distractors (vs. central) significantly induced greater P3 magnitudes, revealing that higher amplitudes of both high‐load and peripheral conditions may result from a greater synchronization of brain resources (Jo et al., 2016). On the other hand, a potential explanation from an attentional resources perspective for the parietal P3 component may be the fact that its amplitude is related to the intensity of attentional processing, suggested by previous findings that P3 amplitudes depend on the capacity for processing task‐relevant stimuli and the increasing consolidation of attentional resources for task‐induced potential amplitudes as resource demands grow (Kok, 2001; Martens et al., 2006). Consistent with expectations, P3 elegantly complements behavioral measures of attentional resources, demonstrating that the attentional resource differences between high‐ and low‐load conditions were reflected by both neural and behavioral operationalizations, as well as providing a potential explanation for behavioral differences in eccentricity.

We then related the observed electrophysiological changes in attention (i.e., P3) to behavioral performance. Studies revealed that people with high MAAS scores, more specifically, those with an advantage in voluntary attentional control, had an enhanced attentional processing intensity reflected by higher P3 amplitudes (Brown et al., 2015; Quaglia et al., 2016). We observed an analogous outcome, that is, a significant positive correlation between P3 amplitudes and MAAS scores. This is an interesting finding because it not only reveals that the ability to focus attention is more likely to correlate with the intensity of attentional processing regardless of load manipulations but also suggests the possibility that the attentional processing of task‐relevant stimuli can be enhanced or assessed through purposeful daily training that improves one's ability to control attention. In addition, there was a significant negative correlation between the P3 amplitude and the error rate in high‐load conditions. It seems that individuals with higher P3 amplitudes in attentional processing were better adapted to the current task due to their likely stronger processing of task goals and greater synchronization of brain resources, especially when their attentional resources were challenged (i.e., high load), leading to better task performance overall (Gan et al., 2020). This trend was sensitive to load manipulations, possibly because the high‐load condition was more attentional‐resource consuming and distraction processing was less effective, in which case the error rates may better characterize the task processing performance of individuals (for a comparable explanation of P3 being sensitive to increasing load, see Bidet‐Caulet et al., 2015). Interestingly, we found a positive correlation between the compatibility effect of P3 latency and behavior on a time scale, primarily driven by the performance in the high‐load condition. The similar pattern of this trend being more pronounced when attentional demands increase again reflects the sensitivity of P3 as a signature of attentional capture to load manipulations, which may be an aspect to be considered in subsequent studies. Overall, in our study, the parietal lobe, as a hub node in the executive function network, provided a possible EEG measure of attention, known as P3, which effectively reflected processing outcomes in conditions with higher cognitive domains where the top‐down mechanisms modulated the bottom‐up mechanisms, consistent with the behavioral findings of attention modulation.

There were several limitations in the current study. First, although we tried to avoid or balance as many potentially irrelevant variables as possible, the impact of this balance on ERPs was difficult to assess (e.g., the balance of cortical representations between peripheral and central distractors, additional interference caused by filler items). Hence, we interpreted the relevant results with caution and expect more support from future studies. Second, we focused more on the temporal dimension of modulation in the later selective processing and explored correspondences between behavior and ERP findings. Future studies are encouraged to include other approaches, such as rhythmic oscillations and functional brain connectivity to address attentional load modulation from different perspectives. Third, our study considered male participants to eliminate potential confounding effects. To generalize these findings, we suggest that future studies consider a broader range of population attributes (e.g., females, attention deficit). In addition, given that life experiences, such as gaming, may have varying effects on attention, future research could consider these potential factors and explore whether different levels of proficiency in the use of cognitive resources may respond differently to load manipulation. Fourth, future studies could apply the paradigm of this experiment and further improve it according to their own experimental purposes, for example, by maximizing the number of load manipulations and increasing the number of trials per condition. Fifth, the present study revealed the influences of load on attentional resources and EEG markers. However, attention may include diverse attentional subprocesses, and it would be enlightening to explore the effects of load on different attentional mechanisms (e.g., natural condition) combined with more dimensional techniques, such as fMRI.

## 5 CONCLUSION

This study explored the processing of selective attention modulated by perceptual load during EEG recordings, revealed the specific attentional patterns of P2 and P3 components in terms of late attentional filtering and overall resource management, and highlighted the underlying role of eccentricity in attention. These findings further the understanding of neural mechanisms in favor of perceptual load theory by providing preliminary evidence for attention quantification and perceptual load modulation based on ERP features.

## CONFLICT OF INTEREST STATEMENT

All authors claim that there are no conflicts of interest.

### PEER REVIEW

The peer review history for this article is available at https://publons.com/publon/10.1002/brb3.2907.

## Supporting information

Supp Information

## Data Availability

Some or all data or code generated or used are available from the corresponding author on reasonable request.

## References

[brb32907-bib-0001] Beck, D. M. , & Lavie, N. (2005). Look here but ignore what you see: Effects of distractors at fixation. Journal of Experimental Psychology: Human Perception and Performance, 31(3), 592–607. 15982133 10.1037/0096-1523.31.3.592

[brb32907-bib-0002] Bidet‐Caulet, A. , Bottemanne, L. , Fonteneau, C. , Giard, M. ‐H. , & Bertrand, O. (2015). Brain dynamics of distractibility: Interaction between top‐down and bottom‐up mechanisms of auditory attention. Brain Topography, 28(3), 423–436. 24531985 10.1007/s10548-014-0354-x

[brb32907-bib-0003] Bisley, J. W. , & Goldberg, M. E. (2010). Attention, intention, and priority in the parietal lobe. Annual Review of Neuroscience, 33, 1–21. 10.1146/annurev-neuro-060909-152823 PMC3683564 20192813

[brb32907-bib-0004] Brockhoff, L. , Schindler, S. , Bruchmann, M. , & Straube, T. (2022). Effects of perceptual and working memory load on brain responses to task‐irrelevant stimuli: Review and implications for future research. Neuroscience & Biobehavioral Reviews, 135, 104580. 35189162 10.1016/j.neubiorev.2022.104580

[brb32907-bib-0005] Brown, K. W. , Creswell, J. D. , & Ryan, R. M. (2015). Handbook of mindfulness: Theory, research, and practice. Guilford Publications.

[brb32907-bib-0006] Brown, K. W. , & Ryan, R. M. (2003). The benefits of being present: Mindfulness and its role in psychological well‐being. Journal of Personality and Social Psychology, 84(4), 822–848. 12703651 10.1037/0022-3514.84.4.822

[brb32907-bib-0007] Brown, K. W. , West, A. M. , Loverich, T. M. , & Biegel, G. M. (2011). Assessing adolescent mindfulness: Validation of an Adapted Mindful Attention Awareness Scale in adolescent normative and psychiatric populations. Psychological Assessment, 23, 1023–1033. 21319908 10.1037/a0021338

[brb32907-bib-0008] Calvo, M. G. , & Castillo, M. D. (2005). Foveal vs. parafoveal attention‐grabbing power of Threat‐related information. Experimental Psychology, 52(2), 150–162. 15850162 10.1027/1618-3169.52.2.150

[brb32907-bib-0009] Cao, R. , Cao, G. , & Liu, P. (2020). Increasing perceptual salience diminishes the motor interference effect from dangerous objects. Frontiers in Psychology, 11, 580. 32292380 10.3389/fpsyg.2020.00580 PMC7118218

[brb32907-bib-0010] Carlson, J. M. (2021). A systematic review of event‐related potentials as outcome measures of attention bias modification. Psychophysiology, 58(6), e13801. 33682161 10.1111/psyp.13801

[brb32907-bib-0011] Cheyne, J. A. , Carriere, J. S. A. , & Smilek, D. (2006). Absent‐mindedness: Lapses of conscious awareness and everyday cognitive failures. Consciousness and Cognition, 15(3), 578–592. 16427318 10.1016/j.concog.2005.11.009

[brb32907-bib-0012] Correll, J. , Urland, G. R. , & Ito, T. A. (2006). Event‐related potentials and the decision to shoot: The role of threat perception and cognitive control. Journal of Experimental Social Psychology, 42(1), 120–128.

[brb32907-bib-0013] Daniel, P. M. , & Whitteridge, D. (1961). The representation of the visual field on the cerebral cortex in monkeys. The Journal of Physiology, 159(2), 203–221. 13883391 10.1113/jphysiol.1961.sp006803 PMC1359500

[brb32907-bib-0014] Delorme, A. , & Makeig, S. (2004). EEGLAB: An open source toolbox for analysis of single‐trial EEG dynamics including independent component analysis. Journal of Neuroscience Methods, 134(1), 9–21. 15102499 10.1016/j.jneumeth.2003.10.009

[brb32907-bib-0015] Doallo, S. , Holguín, S. R. , & Cadaveira, F. (2006). Attentional load affects automatic emotional processing: Evidence from event‐related potentials. NeuroReport, 17(17), 1797–801. 17164667 10.1097/01.wnr.0000246325.51191.39

[brb32907-bib-0016] Doucet, C. , & Stelmack, R. M. (1999). The effect of response execution on P3 latency, reaction time, and movement time. Psychophysiology, 36(3), 351–363. 10352559 10.1017/s0048577299980563

[brb32907-bib-0017] Faul, F. , Erdfelder, E. , Buchner, A. , & Lang, A. ‐G. (2009). Statistical power analyses using G*Power 3.1: Tests for correlation and regression analyses. Behavior Research Methods, 41(4), 1149–1160. 19897823 10.3758/BRM.41.4.1149

[brb32907-bib-0018] Federmeier, K. D. , Mai, H. , & Kutas, M. (2005). Both sides get the point: Hemispheric sensitivities to sentential constraint. Memory & Cognition, 33(5), 871–886. 16383175 10.3758/bf03193082

[brb32907-bib-0019] Fritzsche, A.‐S. , Stahl, J. , & Gibbons, H. (2011). An ERP study of target competition: Individual differences in functional impulsive behavior. International Journal of Psychophysiology, 81(1), 12–21. 21510982 10.1016/j.ijpsycho.2011.03.014

[brb32907-bib-0020] Fu, S. , Fedota, J. , Greenwood, P. M. , & Parasuraman, R. (2010). Early interaction between perceptual load and involuntary attention: An event‐related potential study. Neuroscience Letters, 468(1), 68–71. 19874869 10.1016/j.neulet.2009.10.065 PMC2796074

[brb32907-bib-0021] Gan, X. , Yao, Y. , Liu, H. , Zong, X. , Cui, R. , Qiu, N. , Xie, J. , Jiang, D. , Ying, S. , Tang, X. , Dong, L. , Gong, D. , Ma, W. , & Liu, T. (2020). Action real‐time strategy gaming experience related to increased attentional resources: An attentional blink study. Frontiers in Human Neuroscience, 14, 101. 32341688 10.3389/fnhum.2020.00101 PMC7163005

[brb32907-bib-0022] Geden, M. , Staicu, A.‐M. , & Feng, J. (2018). The impacts of perceptual load and driving duration on mind wandering in driving. Special Issue on Everyday Driving, 57, 75–83.

[brb32907-bib-0023] Giesbrecht, B. , Woldorff, M. G. , Song, A. W. , & Mangun, G. R. (2003). Neural mechanisms of top‐down control during spatial and feature attention. NeuroImage, 19(3), 496–512. 12880783 10.1016/s1053-8119(03)00162-9

[brb32907-bib-0024] Green, C. S. , & Bavelier, D. (2003). Action video game modifies visual selective attention. Nature, 423(6939), 534–537. 12774121 10.1038/nature01647

[brb32907-bib-0025] Green, C. S. , & Bavelier, D. (2006). Effect of action video games on the spatial distribution of visuospatial attention. Journal of Experimental Psychology: Human Perception and Performance, 32(6), 1465–1478. 17154785 10.1037/0096-1523.32.6.1465 PMC2896828

[brb32907-bib-0026] Handy, T. C. , & Mangun, G. R. (2000). Attention and spatial selection: Electrophysiological evidence for modulation by perceptual load. Perception & Psychophysics, 62(1), 175–186. 10703265 10.3758/bf03212070

[brb32907-bib-0027] Handy, T. C. , Soltani, M. , & Mangun, G. R. (2001). Perceptual load and visuocortical processing: Event‐related potentials reveal sensory‐level selection. Psychological Science, 12(3), 213–218. 11437303 10.1111/1467-9280.00338

[brb32907-bib-0028] Harris, A. M. , Eayrs, J. O. , & Lavie, N. (2019). The effect of perceptual load on gaze and EEG signals in multi‐target visual search with free eye‐movements. Journal of Vision, 19(10), 273–273.

[brb32907-bib-0029] Herrmann, C. S. , & Knight, R. T. (2001). Mechanisms of human attention: Event‐related potentials and oscillations. Neuroscience & Biobehavioral Reviews, 25(6), 465–476. 11595268 10.1016/s0149-7634(01)00027-6

[brb32907-bib-0030] Jo, H.‐G. , Schmidt, S. , Inacker, E. , Markowiak, M. , & Hinterberger, T. (2016). Meditation and attention: A controlled study on long‐term meditators in behavioral performance and event‐related potentials of attentional control. International Journal of Psychophysiology, 99, 33–39. 26659014 10.1016/j.ijpsycho.2015.11.016

[brb32907-bib-0031] Kang, H. (2021). Sample size determination and power analysis using the G*Power software. Journal of Educational Evaluation for Health Professions, 18, 17. 34325496 10.3352/jeehp.2021.18.17 PMC8441096

[brb32907-bib-0032] Keith, J. R. , Blackwood, M. E. , Mathew, R. T. , & Lecci, L. B. (2017). Self‐reported mindful attention and awareness, go/no‐go response‐time variability, and attention‐deficit hyperactivity disorder. Mindfulness, 8(3), 765–774. 28458727 10.1007/s12671-016-0655-0 PMC5407284

[brb32907-bib-0033] Kiesel, A. , Miller, J. , Jolicœur, P. , & Brisson, B. (2008). Measurement of ERP latency differences: A comparison of single‐participant and jackknife‐based scoring methods. Psychophysiology, 45(2), 250–274. 17995913 10.1111/j.1469-8986.2007.00618.x

[brb32907-bib-0034] Koelstra, S. , Muhl, C. , & Patras, I. (2009). EEG analysis for implicit tagging of video data. 2009 3rd International Conference on Affective Computing and Intelligent Interaction and Workshops. IEEE.

[brb32907-bib-0035] Kok, A. (2001). On the utility of P3 amplitude as a measure of processing capacity. Psychophysiology, 38(3), 557–577. 11352145 10.1017/s0048577201990559

[brb32907-bib-0036] Koroma, M. , Lacaux, C. , Andrillon, T. , Legendre, G. , Léger, D. , & Kouider, S. (2020). Sleepers selectively suppress informative inputs during rapid eye movements. Current Biology, 30(12), 2411–2417.e3. 32413310 10.1016/j.cub.2020.04.047

[brb32907-bib-0037] Lavie, N. (1995). Perceptual load as a necessary condition for selective attention. Journal of Experimental Psychology: Human Perception and Performance, 21(3), 451–468. 7790827 10.1037//0096-1523.21.3.451

[brb32907-bib-0038] Lavie, N. (2005). Distracted and confused?: Selective attention under load. Trends in Cognitive Sciences, 9(2), 75–82. 15668100 10.1016/j.tics.2004.12.004

[brb32907-bib-0039] Lavie, N. (2010). Attention, distraction, and cognitive control under load. Current Directions in Psychological Science, 19(3), 143–148.

[brb32907-bib-0040] Lavie, N. , Hirst, A. , de Fockert, J. W. , & Viding, E. (2004). Load theory of selective attention and cognitive control. Journal of Experimental Psychology: General, 133(3), 339–354. 15355143 10.1037/0096-3445.133.3.339

[brb32907-bib-0041] Li, F. , Chen, B. , Li, H. , Zhang, T. , Wang, F. , Jiang, Y. , Li, P. , Ma, T. , Zhang, R. , Tian, Y. , Liu, T. , Guo, D. , Yao, D. , & Xu, P. (2016). The time‐varying networks in P300: A task‐evoked EEG study. IEEE Transactions on Neural Systems and Rehabilitation Engineering, 24(7), 725–733. 26849870 10.1109/TNSRE.2016.2523678

[brb32907-bib-0042] Lin, Y. , Fisher, M. E. , & Moser, J. S. (2018). Clarifying the relationship between mindfulness and executive attention: A combined behavioral and neurophysiological study. Social Cognitive and Affective Neuroscience, 14(2), 205–215. 10.1093/scan/nsy113 PMC6374600 30535128

[brb32907-bib-0043] Lopez‐Calderon, J. , & Luck, S. J. (2014). ERPLAB: An open‐source toolbox for the analysis of event‐related potentials. Frontiers in Human Neuroscience, 8, 213. 24782741 10.3389/fnhum.2014.00213 PMC3995046

[brb32907-bib-0044] Luck, S. J. , & Hillyard, S. A. (1990). Electrophysiological evidence for parallel and serial processing during visual search. Perception & Psychophysics, 48(6), 603–617. 2270192 10.3758/bf03211606

[brb32907-bib-0045] Luck, S. J. , & Hillyard, S. A. (1994). Electrophysiological correlates of feature analysis during visual search. Psychophysiology, 31(3), 291–308. 8008793 10.1111/j.1469-8986.1994.tb02218.x

[brb32907-bib-0046] Martens, S. , Elmallah, K. , London, R. , & Johnson, A. (2006). Cuing and stimulus probability effects on the P3 and the AB. Acta Psychologica, 123(3), 204–218. 17099955 10.1016/j.actpsy.2006.01.001

[brb32907-bib-0047] Maylor, E. A. , & Lavie, N. (1998). The influence of perceptual load on age differences in selective attention. Psychology and Aging, 13(4), 563–573. 9883457 10.1037//0882-7974.13.4.563

[brb32907-bib-0048] Neokleous, K. , Shimi, A. , & Avraamides, M. N. (2016). Modeling the effects of perceptual load: Saliency, competitive interactions, and top‐down biases. Frontiers in Psychology, 7, 1. 26858668 10.3389/fpsyg.2016.00001 PMC4726798

[brb32907-bib-0049] Nikolaev, A. R. , Ziessler, M. , Dimova, K. , & van Leeuwen, C. (2008). Anticipated action consequences as a nexus between action and perception: Evidence from event‐related potentials. Biological Psychology, 78(1), 53–65. 18289769 10.1016/j.biopsycho.2007.12.010

[brb32907-bib-0050] Perrone‐Bertolotti, M. , El Bouzaïdi Tiali, S. , Vidal, J. R. , Petton, M. , Croize, A. C. , Deman, P. , Rheims, S. , Minotti, L. , Bhattacharjee, M. , Baciu, M. , Kahane, P. , & Lachaux, J. P. (2020). A real‐time marker of object‐based attention in the human brain. A possible component of a “gate‐keeping mechanism” performing late attentional selection in the ventro‐lateral prefrontal cortex. NeuroImage, 210, 116574. 31981780 10.1016/j.neuroimage.2020.116574

[brb32907-bib-0051] Pfefferbaum, A. , Ford, J. M. , Roth, W. T. , & Kopell, B. S. (1980). Age‐related changes in auditory event‐related potentials. Electroencephalography and Clinical Neurophysiology, 49(3), 266–276. 6158403 10.1016/0013-4694(80)90221-7

[brb32907-bib-0052] Pinsk, M. A. , Doniger, G. M. , & Kastner, S. (2004). Push‐Pull mechanism of selective attention in human extrastriate cortex. Journal of Neurophysiology, 92(1), 622–629. 14973320 10.1152/jn.00974.2003

[brb32907-bib-0053] Polich, J. (2012). Neuropsychology of P300. In Oxford Library of Psychology. The Oxford handbook of event‐related potential components pp. 159–188. Oxford University Press.

[brb32907-bib-0054] Proksch, J. , & Bavelier, D. (2002). Changes in the spatial distribution of visual attention after early deafness. Journal of Cognitive Neuroscience, 14(5), 687–701. 12167254 10.1162/08989290260138591

[brb32907-bib-0055] Quaglia, J. T. , Goodman, R. J. , & Brown, K. W. (2016). Trait mindfulness predicts efficient top‐down attention to and discrimination of facial expressions: Mindfulness in socioemotional contexts. Journal of Personality, 84(3), 393–404. 25676934 10.1111/jopy.12167

[brb32907-bib-0056] Rees, G. , Frith, C. D. , & Lavie, N. (1997). Modulating irrelevant motion perception by varying attentional load in an unrelated task. Science, 278(5343), 1616–1619. 9374459 10.1126/science.278.5343.1616

[brb32907-bib-0057] Rovamo, J. , & Virsu, V. (1979). An estimation and application of the human cortical magnification factor. Experimental Brain Research, 37(3), 495–510. 520439 10.1007/BF00236819

[brb32907-bib-0058] Sawaki, R. , & Luck, S. J. (2010). Capture versus suppression of attention by salient singletons: Electrophysiological evidence for an automatic attend‐to‐me signal. Attention, Perception, & Psychophysics, 72(6), 1455–1470. 10.3758/APP.72.6.1455 PMC3705921 20675793

[brb32907-bib-0059] Schindler, S. , Bruchmann, M. , Gathmann, B. , Moeck, R. , & Straube, T. (2021). Effects of low‐level visual information and perceptual load on P1 and N170 responses to emotional expressions. Cortex; A Journal Devoted to the Study of the Nervous System and Behavior, 136, 14–27. 33450599 10.1016/j.cortex.2020.12.011

[brb32907-bib-0060] Schubö, A. (2009). Salience detection and attentional capture. Psychological Research, 73(2), 233–243. 19066945 10.1007/s00426-008-0215-x

[brb32907-bib-0061] Tian, Y. , & Yao, D. (2013). Why do we need to use a zero reference? Reference influences on the ERPs of audiovisual effects: Reference influence on ERPs. Psychophysiology, 50(12), 1282–1290. 23941085 10.1111/psyp.12130

[brb32907-bib-0062] Tyndall, I. , Ragless, L. , & O'Hora, D. (2018). Effects of perceptual load and socially meaningful stimuli on crossmodal selective attention in autism spectrum disorder and neurotypical samples. Consciousness and Cognition, 60, 25–36. 29522997 10.1016/j.concog.2018.02.006

[brb32907-bib-0063] Watson, P. , Pearson, D. , Theeuwes, J. , Most, S. B. , & Le Pelley, M. E. (2020). Delayed disengagement of attention from distractors signalling reward. Cognition, 195, 104125. 31751815 10.1016/j.cognition.2019.104125

[brb32907-bib-0064] Witt, S. T. , van Ettinger‐Veenstra, H. , Salo, T. , Riedel, M. C. , & Laird, A. R. (2021). What executive function network is that? An image‐based meta‐analysis of network labels. Brain Topography, 34(5), 598–607. 33970388 10.1007/s10548-021-00847-z

[brb32907-bib-0065] Yao, D. (2001). A method to standardize a reference of scalp EEG recordings to a point at infinity. Physiological Measurement, 22(4), 693–711. 11761077 10.1088/0967-3334/22/4/305

