# Supplementary material for: Event‐related potential patterns of selective attention modulated by perceptual load
Source: Brain Behav. 2023 Feb 14;13(3):e2907. doi: 10.1002/brb3.2907 (PMC10013938; doi:10.1002/brb3.2907)
Supplement: Supplementary file 1 — Supp Information [file BRB3-13-e2907-s001.docx]

## The supplementary information of event-related potential (ERP) patterns of selective attention modulated by perceptual load

Zhuo Chen^1 †^, Yun Qin^1 †^, Maoqin Peng^2^, Wei Zhao^1^, Xuqian Shi^1^, Danwei Lai^1^, Erwei Yin^3^, Ye Yan^3^, Dezhong Yao^1 *^ and Tiejun Liu^1 *^

*^1^MOE Key Lab for Neuroinformation, School of Life Science and Technology,* *University of Electronic Science and Technology of China, Chengdu 611731, China*

*^2^ College of Electronic Engineering, Chengdu University of Information Technology, Chengdu 610225, China*

*^3^**The Defense Innovation Institute, Academy of Military Sciences, Beijing 100071, China*

**Table 1.** Responses of participants (n = 31) for mean reaction times (RTs, in Milliseconds) and error rates as a function of perceptual load, distractor compatibility, and distractor eccentricity.

| Distractor eccentricity | Distractor compatibility | | | | |
| --- | --- | --- | --- | --- | --- |
|  | Compatible | |  | Incompatible | |
|  | RT | % error |  | RT | % error |
| Center |  |  |  |  |  |
| Low load | 672 (9) | 3 (1) |  | 688 (8) | 7 (1) |
| High load | 879 (18) | 21 (2) |  | 896 (19) | 27 (2) |
| Periphery |  |  |  |  |  |
| Low load | 678 (9) | 3 (1) |  | 699 (9) | 6 (1) |
| High load | 898 (20) | 21 (2) |  | 892 (18) | 23 (2) |

*Note.* Numbers in parentheses indicates standard errors.

**Table 2.** Descriptive P2 data at Fz electrode for both 50% area latency in milliseconds and mean amplitude in μV at the same time period as a function of perceptual load, distractor compatibility, and distractor eccentricity.

| Distractor eccentricity | Distractor compatibility | | | | |
| --- | --- | --- | --- | --- | --- |
|  | Compatible | |  | Incompatible | |
|  | Latency | Amplitude |  | Latency | Amplitude |
| Center |  |  |  |  |  |
| Low load | 225.62 (1.85) | 0.94  (0.32) |  | 221.96 (2.85) | 1.49  (0.32) |
| High load | 223.11 (2.29) | 0.75  (0.31) |  | 222.22 (2.06) | 1.04  (0.36) |
| Periphery |  |  |  |  |  |
| Low load | 225.93 (1.87) | 1.03  (0.31) |  | 225.65 (2.89) | 0.95  (0.29) |
| High load | 221.55 (1.89) | 0.94  (0.37) |  | 220.10 (2.04) | 1.16  (0.35) |

*Note.* Numbers in parentheses indicates standard errors.

**Table 3.** Descriptive P3 data at Pz electrode for both 50% area latency in milliseconds and mean amplitude in μV at the same time period as a function of perceptual load, distractor compatibility, and distractor eccentricity.

| Distractor eccentricity | Distractor compatibility | | | | |
| --- | --- | --- | --- | --- | --- |
|  | Compatible | |  | Incompatible | |
|  | Latency | Amplitude |  | Latency | Amplitude |
| Center |  |  |  |  |  |
| Low load | 285.01  (3.22) | 0.19  (0.52) |  | 290.49  (2.65) | 0.61  (0.45) |
| High load | 289.22  (2.40) | 1.06  (0.53) |  | 289.86  (2.62) | 0.65  (0.52) |
| Periphery |  |  |  |  |  |
| Low load | 287.79  (2.13) | 1.05  (0.51) |  | 288.46  (2.05) | 1.07  (0.47) |
| High load | 290.11  (1.93) | 1.74  (0.51) |  | 286.10  (2.15) | 1.59  (0.53) |

*Note.* Numbers in parentheses indicates standard errors.

**Results of a three-way ANOVA of the P2 latency at F3 and F4:**

We further performed a three-way ANOVA similar to Fz with 2 (perceptual load: low vs. high) × 2 (distractor eccentricity: central vs. peripheral) × 2 (compatibility: compatible vs. incompatible) for P2 latencies at F3 and F4, respectively.

Results for electrode F3:

There was a main effect of load [*F*(1, 30) = 8.71, *p* =.006, $\eta_{p}^{2}$ = 0.23] (low load: 223.43 ms ± 2.19; high load: 221.02 ms ± 1.81). No other main effects (eccentricity: *p* =.639; compatibility: *p* =.358) or interaction effects (compatibility × eccentricity: *p* =.696; compatibility × load: *p* =.413; load × eccentricity: *p* =.229; compatibility × eccentricity × load: *p* =.841) were significant.

Results for electrode F4:

There were a main effect of compatibility [*F*(1, 30) = 5.47, *p* =.026, $\eta_{p}^{2}$ = 0.15] (compatible: 224.27 ms ± 1.76; incompatible: 222.00 ms ± 2.06) and a marginally significant main effect of load [*F*(1, 30) = 3.89, *p* =.058, $\eta_{p}^{2}$ = 0.12] (low load: 224.21 ms ± 2.07; high load: 222.05 ms ± 1.78). Other main or interaction effects (eccentricity: *p* =.099; compatibility × eccentricity: *p* =.247; compatibility × load: *p* =.616; load × eccentricity: *p* =.537; compatibility × eccentricity × load: *p* =.244) did not reach statistical significance.


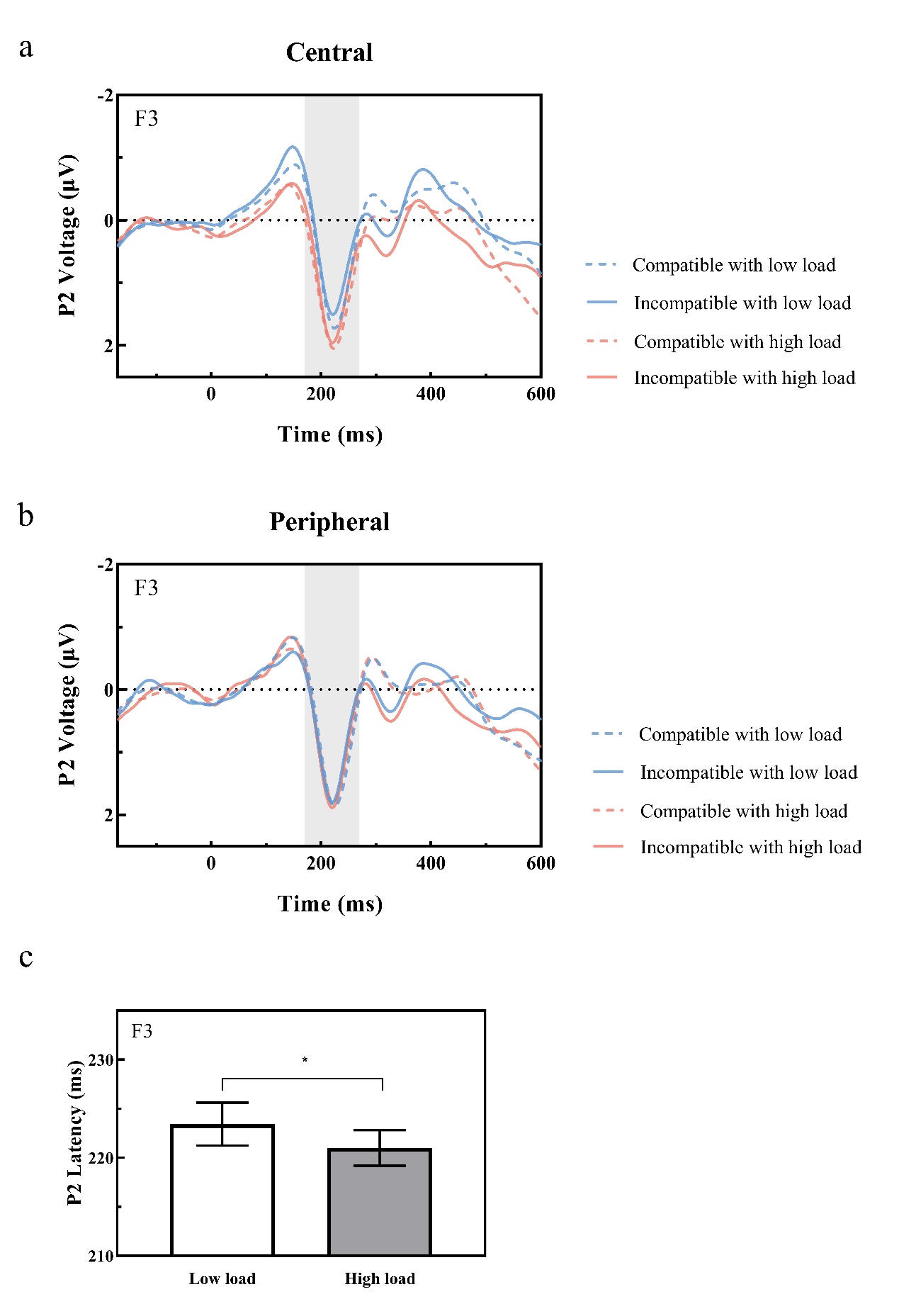


**Fig. S1.** P2 data for central distractors (**a**) and peripheral distractors (**b**) under differential load levels at electrode F3. **c**, The high load induced a significantly shorter latency than the low load. Error bars refer to SEM, **p* <.05, ***p* <.001.


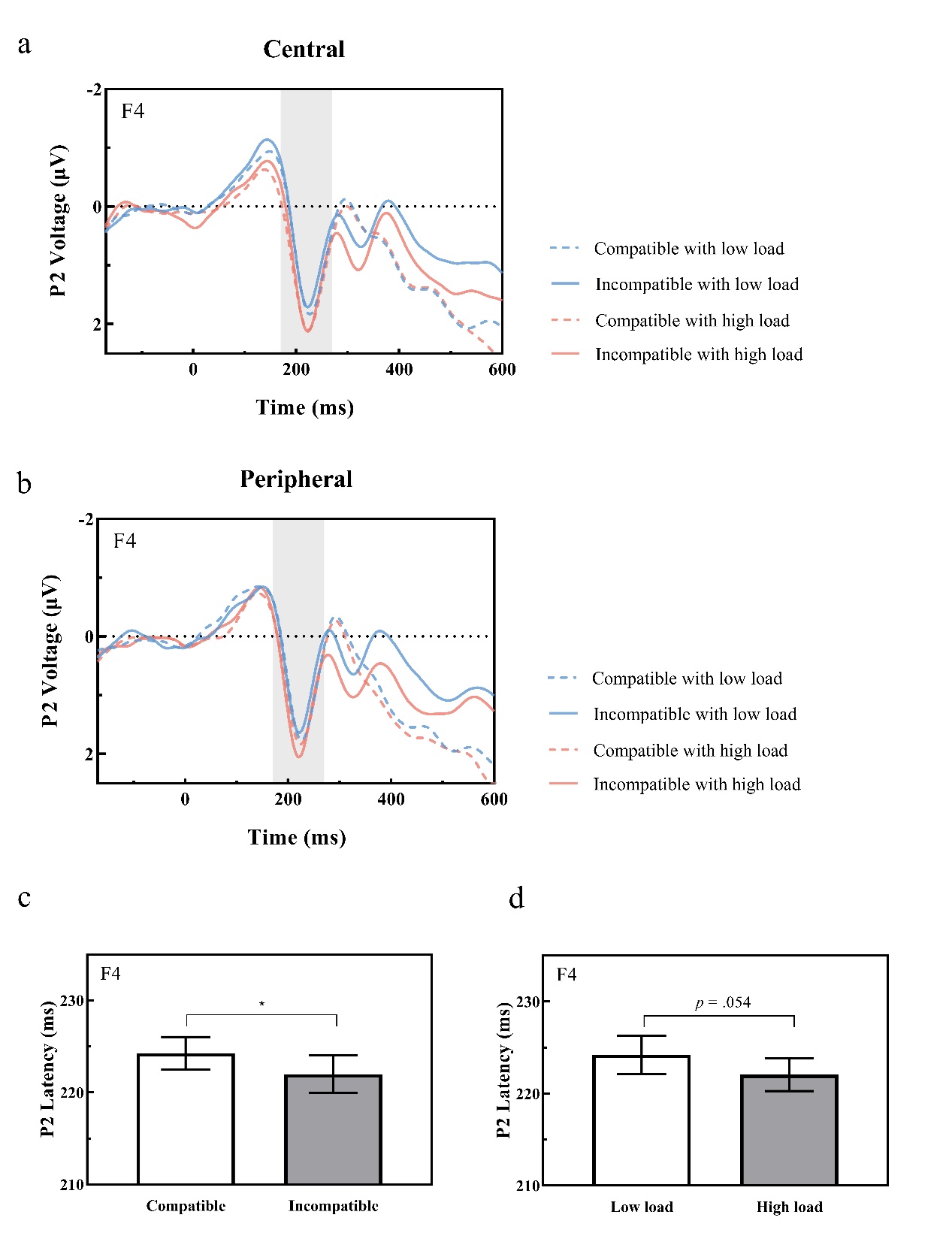


**Fig. S2.** P2 data for central distractors (**a**) and peripheral distractors (**b**) under differential load levels at electrode F4. **c**, The differences between compatibility and incompatibility trials. **d**, The high load trials reflected a tendency to have shorter latency than the low load. Error bars refer to SEM, **p* <.05, ***p* <.001.
